# Strategies to Increase Willingness to Receive a COVID-19 Vaccine among Nursing Home Staff

**DOI:** 10.3390/idr15010004

**Published:** 2022-12-31

**Authors:** Lisa S. van Tol, Wendy Meester, Monique A. A. Caljouw, Wilco P. Achterberg

**Affiliations:** 1 University Network for the Care Sector South Holland, Leiden University Medical Centre, P.O. Box 9600, 2300 RC Leiden, The Netherlands; 2 Department of Public Health and Primary Care, Leiden University Medical Centre, P.O. Box 9600, 2300 RC Leiden, The Netherlands

**Keywords:** COVID-19, vaccination, nursing homes, staff

## Abstract

Background: Nursing home (NH) staff and residents have been prioritized to receive COVID-19 vaccinations. However, NH staff have been hesitant. This study explored what strategies were used to overcome this hesitancy and which of these were found to be important by NH staff to increase their willingness to take a COVID-19 vaccine. Methods: This study employed a sequential exploratory qualitative design. The COVID-19 MINUTES study aimed to describe the challenges presented by, responses to, and impact of the COVID-19 pandemic in NHs. The minutes of COVID-19 outbreak teams (COTs) in Dutch long-term care organizations (*n* = 41) were collected and coded using content analysis. Textual units from December 2020 to April 2021 that regarded strategies to increase staff’s vaccination willingness (*n* = 67) were selected. Subsequently, to validate these data, two panels of NH healthcare workers (HCWs) and policy workers (PWs) (*n* = 8) selected, discussed, and ranked the strategies that they found to be important using a modified nominal group technique. Results: The strategies described in the minutes included financial reimbursements, personal contact, story sharing, logistics support, role models, visual information, and written information. Except for financial reimbursement, all these strategies were considered important or very important by the panel participants. Some organizations combined multiple strategies. Conclusion: The strategies that were found important in combination may be used more broadly and should be developed further with the involvement of HCWs.

## 1. Introduction

Internationally, almost half of the deaths linked to COVID-19 and many of the most severe cases of COVID-19 have occurred among nursing home (NH) and other long-term care (LTC) residents [1,2]. In addition, the staff who care for and have close contact with NH residents have increased risks of COVID-19 morbidity and mortality [3]. Fortunately, COVID-19 vaccines are now available. These vaccines have been shown to be highly effective against infection in the general population (86%) and older persons (84%), and the most effective among healthcare workers (HCWs) (95%) [4]. COVID-19 vaccination is also highly effective against mortality (>90%) among the Dutch general population and older long-term care users [5]. The vaccination of NH staff against COVID-19 decreases their risk of becoming infected [6] and transmitting the virus [7,8] and can reduce the associated high numbers of absenteeism and staff shortages [9]. In addition, the willingness of NH staff to get a COVID-19 vaccine may reflect on the willingness of NH residents to do so, as they regard staff as a reliable source of information [10]. Thus, it is important to stimulate the uptake of COVID-19 vaccines among NH staff.

Most high income countries (HICs) have prioritized NH staff and residents to receive COVID-19 vaccines [11]. However, internationally, only half of HCWs have been willing to receive a COVID-19 vaccine [12]. The willingness among HCWs in HICs to be vaccinated varied from 33 to 77% [10]. Between 38 and 83% of staff in HIC NHs had actually received a first dose of the vaccination against COVID-19 by January–March 2021 [13,14]. According to the World Health Organization, vaccine hesitancy is one of the ten greatest threats to global health [15].

Common reasons for hesitation towards COVID-19 vaccination among NH staff and other HCWs include concerns about side effects [16,17,18]; a lack of confidence in the safety, adequate testing, and effectiveness of COVID-19 vaccines [9,17,19,20]; the perceived low severity and risk of COVID-19 [19]; a lack of information about the vaccine [18,19]; distrust in the government [9]; and religion [16]. Except for side effects and a lack of information, similar reasons for hesitation were commonly reported among international general populations [21,22]. Moreover, vaccine hesitancy among general populations has been associated with sociodemographic characteristics, including age, gender, education, and occupation [21].

Thus, there is a great need for the development of strategies to stimulate the uptake of vaccination or increase staff knowledge and awareness about vaccines. To overcome low vaccination rates, a few countries have mandated COVID-19 vaccination for NH staff [11]. However, before mandatory vaccination is justified, less invasive strategies to increase staff willingness to get a COVID-19 vaccine voluntarily should be considered [23,24]. Little research has been done on such strategies. Therefore, the aims of this study were to explore what strategies were used by COVID-19 outbreak teams to increase the willingness of staff to get a COVID-19 vaccine in Dutch NHs and to explore which of these strategies were considered important by NH staff.

## 2. Materials and Methods

### 2.1. Design

The present study employed a sequential exploratory qualitative design [25]. The first part of this study is embedded in the COVID-19 Management in Nursing Homes by Outbreak Teams (MINUTES) study [26]. The objective of the MINUTES study was to describe the challenges presented by, responses to, and impact of the COVID-19 pandemic in NHs. The minutes of COVID-19 outbreak teams (COTs) in LTC organizations were collected from March 2020 to October 2021. Strategies described to increase the willingness of NH staff to get a COVID-19 vaccine were selected from these data. The second part of our investigation entailed a panel study in which NH staff prioritized important COVID-19 measures. In this panel study, we made use of a nominal group technique (NGT) that was modified into an online procedure with a single rating phase. To validate the data from the MINUTES study, panels of NH staff selected, discussed, and rated the strategies they found to be the most important in contributing to increasing in their willingness to receive a COVID-19 vaccine.

### 2.2. Setting

COVID-19 vaccination in the Netherlands started on 6 January 2021 with the vaccination of NH and small-scale living staff [27]. Official numbers for vaccination coverage among NH staff are lacking, as the General Data Protection Regulation [28] does not allow employers to process vaccination status, which is classified as special personal data. 

### 2.3. Data Collection and Analysis

Minutes from central COTs in 41 Dutch LTC organizations were saved and coded in an online CASTOR database [29] using qualitative content analysis [26]. For the present study, we extracted all textual units that were coded within the code ‘vaccination’ from around the start of COVID-19 vaccination (early December (week 49) 2020 to early March (week 9) 2021). Next, we selected textual units that could be interpreted as strategies to increase the willingness of staff to get a COVID-19 vaccine, hereafter called strategies. The types of strategies were inductively coded.

Subsequently, participating LTC organizations that described strategies in their COT minutes were invited by email to participate in panel conversations. The aim was to organize panels of four to seven policy workers (PWs) and four to seven HCWs until reaching data saturation. An online modified NGT [30,31] was performed consisting of three stages: First, before the panel conversation, participants selected the strategy that they found to be most important. To activate their thoughts on the topic, they were provided with a list of textual units from the MINUTES data that illustrated actions by COTs that could be interpreted as strategies (December 2020–early March 2021). Second, during a 1.5 to 2 h online panel conversation via Zoom [32], participants presented their selected strategies to each other. Subsequently, they discussed their ideas. The conversations were guided by a facilitator (WM) and an assistant facilitator who also took minutes and had experience with facilitating online modified NGT panels (LST). The audio from the panel conversations was recorded, partly transcribed verbatim, analyzed using content analysis, and converted into strategy summaries. 

Third, within one week, participants received a form with these summaries by email. They were asked to rate the importance of each strategy on a 5-point Likert scale. In addition, they could provide comments on the summaries as a check for correct interpretation by the researchers. The ratings given were then counted. If multiple boxes were ticked, the lowest value was kept. The strategies from the minutes were validated by comparing and matching their code names with the strategies discussed by the panels.

## 3. Results

### 3.1. Participants

A total of 41 Dutch LTC organizations participated in the MINUTES study. These organizations varied in size from 3 to 70 NH locations, but together represented 563 NHs [26]. All organizations’ COTs were installed between mid-February (week 8) and the end of March (week 13) 2020. From December 2020 to April 2021, 663 textual units in their minutes were coded with the code ‘vaccination’. Of these, 67 textual units included data that could be interpreted as strategies. These data about strategies originated from 21 organizations’ minutes. Out of these 21 organizations, the 11 organizations that discussed the topic most were invited to participate in the panels; however, many staff members rejected participation due to COVID-19-related workload. 

Eight participants representing three organizations were recruited. The first panel consisted of five PWs: one care manager, two policymakers, one human resource advisor, and one quality assurance nurse. Three HCWs participated in the second panel: two nurses and one activity supervisor. The participants’ mean age was 44 years old (SD 11.5) and only one was male. Except for one participant, all were vaccinated or were planning to get vaccinated (April 2021). For two participants, this decision was influenced by their organizations’ strategies (Table 1). According to the panel participants, decisions regarding their organizations’ strategies were made by COTs. The participants provided few comments on the summaries of strategies discussed.

### 3.2. Strategies to Increase the Willingness of Staff to Get a COVID-19 Vaccine

**Figure 1:**
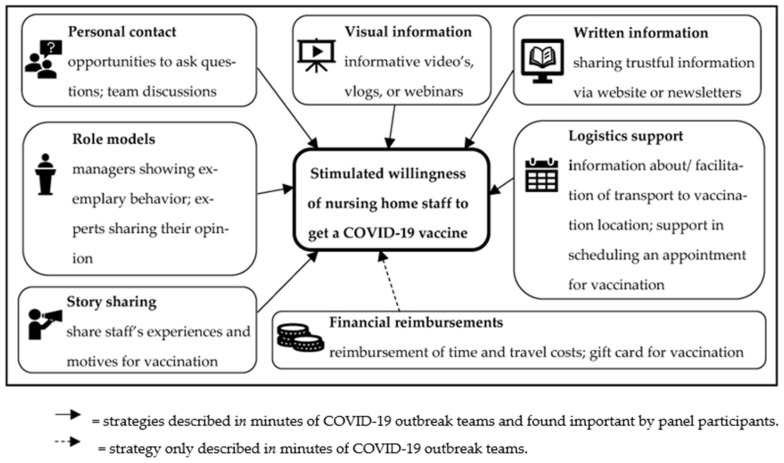
Strategies to stimulate uptake of COVID-19 vaccines by nursing home staff.

Seven types of strategies were identified from the COT minutes: financial reimbursements (*n* = 17 textual units), personal contact (*n* = 14), story sharing (*n* = 3), logistics support (*n* = 8), role models (*n* = 2), visual information (*n* = 5), and written information (*n* = 14). Some COTs combined various strategies (*n* = 4). Apart from financial reimbursements, all strategies were selected to be important and discussed by the panels (Table 2; Figure 1).

#### 3.2.1. Financial Reimbursements

It was frequently described in the minutes that staff could claim time and travel costs to a vaccination location. A few textual units described that staff could apply, confidentially, for a gift card after they were vaccinated. Organizations used this to get insight into vaccination rates: “*Healthcare workers can write two hours of (extra) working time per vaccination. [..] Inform planners in advance that this is approved. In addition, it is important that they know that they have a duty of confidentiality.*” (COT minutes). However, the PW panel discussed the idea that financial reimbursements would not be useful: “*I think a gift card won’t stimulate vaccination willingness. On the contrary, it will drive people away if they feel this is intended to encourage them.*” (quality assurance nurse).

#### 3.2.2. Personal Contact

Some organizations took minutes about introducing telephone numbers, email addresses, and walk-in hours where staff could ask their questions. Others organized team meetings to inform staff about the vaccination program and the importance of vaccination and to allow staff to discuss vaccination with each other and with their supervisors: “*Elderly care physicians already called on all physicians to conducting low-threshold conversations and answering questions*” (COT minutes). All panel participants rated these types of personal contact as important or very important. However, one participant questioned whether staff would discuss personal questions with direct colleagues: “*I haven’t heard of anybody who would prefer to discuss this with someone you have to work with and discuss clients with. That you would suddenly have to discuss your own situation.*” (nurse B).

#### 3.2.3. Story Sharing

A few organizations’ minutes described sharing the personal experiences and motives of some staff members to be vaccinated: “*Two staff members (one physician and one physician assistant) tell colleagues in a video message why they decided to be vaccinated*” (COT minutes). Three PWs rated this strategy as important, because these experiences set a good example and could be used as a conversation starter among staff. 

#### 3.2.4. Logistics Support

A few COTs reported that they shared information about public transport, encouraged car-pooling, or facilitated taxis to vaccination locations. In addition, COTs occasionally assisted staff members in scheduling vaccination appointments, particularly when appointments were fully booked across the country: “*So when you hear from employees that they were unable to schedule an appointment in the first week, point out that this may now be possible*” (COT minutes). The HCWs described travel facilitation as important, but the facilitation of scheduling vaccination appointments remained undiscussed.

#### 3.2.5. Role Models

A few times, COTs considered if it was possible to use members of management teams and client councils or physicians as role models: “*Because of scarcity [in vaccines] it is difficult to for example vaccinate a management team first.*” (COT minutes). Two HCWs felt that the opinion of an external role model was important. One participant mentioned that the expert opinion of this role model was helpful, even though she trusted her employer completely: “*He is an external person, even though he is connected to the organization, that gave me a little push.*” (activity supervisor). 

#### 3.2.6. Visual Information

Multiple COTs described sharing informative videos, making vlogs, or holding organized webinars: “*Preparation vaccination process (incl. communication). Thursday there is a webinar about corona vaccination [..]. Next webinar for all employees will be in January when the schedule is known. In between, another webinar for management and coaches.*” (COT minutes). All panel participants found the use of vlogs and webinars important or very important, because these encouraged conversation, facilitated the visibility of directors while working from home, and made staff feel seen. One HCW preferred visual information over written information: “*people already have a lot to read and newsletters are often long so I think a vlog will be stimulating*” (nurse A).

#### 3.2.7. Written Information

The COT minutes also described sharing factual or trustful information about vaccines via the organization website and (news)letters to staff: “*As soon as reliable information becomes available it will be shared. It is quite complicated to find factual answers to questions among the many opinions*” (COT minutes). Seven participants rated this strategy as important or very important. They argued that with the multitude of conflicting messages on social media, it is important to provide staff with correct information to base their considerations on. One nuance that was discussed was that the amount of information quickly became too much.

## 4. Discussion

This study identified the strategies used by COTs that were found to be important by NH staff to increase their willingness to receive a COVID-19 vaccine. The strategies identified included financial reimbursements, personal contact, story sharing, logistics support, role models, visual information, and written information. Except for financial reimbursements, all were considered important or very important by NH staff members.

Strategies to increase vaccination willingness among NH staff have hardly been studied. However, the literature exploring COVID-19 vaccination among other HCWs is in line with our findings: personal contact with HCWs [20], story sharing [9,17], and information and education [18] have been suggested to improve vaccine uptake. Nevertheless, information overload can reduce trustworthiness and cause confusion. [33]. Furthermore, combining strategies has been associated with higher vaccination coverage among NH staff, while financial reimbursement has not [34].

The uptake of vaccination and other strategies to combat COVID-19 largely depends on human behavior [35,36]. Therefore, behavior change theories should underpin follow-up research focusing on behavior changes in NH staff, including the design, implementation, and evaluation of the effectiveness of improved strategies. The most commonly used behavioral change theories in the literature surrounding communicable [37] and infectious disease outbreaks [35], emergency responses [35], and influenza vaccination among HCWs [38] include the health belief model and theory of planned behavior. The health belief model recommends cues to action that are in line with the strategies identified [39]. According to the theory of planned behavior, attitudes towards COVID-19 vaccination, social pressure to perform the behavior, and the perceived ease of performing the behavior can predict the willingness of individuals to get a COVID-19 vaccine [40]. In this light, a combination of information to increase knowledge, strategies related to personal contact [10], and story sharing or role models [41] have been suggested. In addition, logistics support may add to the perceived ease of getting a vaccine. 

Moreover, staff should be involved [9,20,33] and “bottom-up” communication should be used in the further development of vaccination strategies [20,34]; an American study identified that the use of “frontline champions” in NHs, similar to role models or story sharing, could be perceived as “bottom-up” communication and was associated with higher vaccine coverage [34]. NHs that used top-down messages from inspirational leaders had lower vaccination coverage [34]. 

The most important limitation of this study is the small number of panel participants. During times of crisis and pressing staff shortages, the recruitment of extra panel participants was difficult. Strongly insisting that the scarce staff participate in our study felt inappropriate. However, there was ample variation in participants’ hesitancy profiles: five participants were (willing to be) vaccinated; one participant did not want to take a COVID-19 vaccine (‘skeptic’); and two participants were influenced by their organizations’ strategies. Future research with more adequate numbers of participants may further explore various hesitancy profiles and associated strategies. The literature on COVID-19 hardly takes into account these different profiles. However, one study among nurses in Hong Kong described skeptics as being hard to persuade. They may be convinced by transparent information and trustworthy healthcare authorities. Doubters may be persuaded with easily accessible information, communicating the severity and the risk of contracting the disease, and logistics support [42].

The most important strength of this study is the sequential exploratory design that was used to validate data from the longitudinal qualitative COVID-19 MINUTES study [26] in two panels of NH staff. Except for financial reimbursements, the strategies described by COVID-19 outbreak teams were also considered to be important by nursing home staff. 

In conclusion, this study adds to the literature by identifying strategies that have been used to overcome hesitancy towards COVID-19 vaccines in NH staff. These are personal contact, story sharing, logistics support, role models, visual information, and written information. These strategies should be developed further with the involvement of HCWs and in combination may be used more broadly in order to increase the willingness of NH staff to receive a COVID-19 vaccine. 

## Figures and Tables

**Table 1:** Panel participants.

Position	Organization	Gender	Age (Years)	Work Experience (Years)	(Planning to Get) Vaccinated	Strategy Selected as Most Important	Strategies Influenced Decision
**Policy workers**							
Human resource advisor	A	Female	36	2	No	Visual information	No
Policymaker A	A	Female	62	5	Yes	Written information	No
Policymaker B	B	Female	51	4	Yes	Visual information	No
Quality assurance nurse	B	Female	30	2	Yes	Visual information	Yes
Care manager	C	Male	44	8	Yes	Personal contact	No
**Healthcare workers**							
Nurse A	B	Female	28	1	Yes	*Multiple categories*	No
Nurse B	A	Missing	44	18	Yes	Written information	Yes
Activity supervisor	B	Female	57	20	Yes	*Multiple categories*	No

**Table 2:** Types of strategies described in the MINUTES study and rated by panels of NH staff.

Types of Strategies	MINUTES Data	Rating by Panel Participants of Measures Selected to be Important			
Rated by *n* Panel Participants	Textual Units (*n* = 67)	Very Important	Important	Not Important Nor Unimportant	Unimportant or Very Unimportant
Financial reimbursement (*n* = 0)	17	N/A	N/A	N/A	N/A
Personal contact (*n* = 8)	14	3	5	0	0
Story sharing (*n* = 5: PW ^1^)	3	0	3	2	0
Logistics support (*n* = 3: HCW ^2^)	8	0	2	1	0
Role models (*n* = 3: HCW ^2^)	2	0	2	1	0
Visual information (*n* = 8)	5	2	6	0	0
Written information (*n* = 8)	14	3	4	1	0
Combinations of strategies	4				

^1^ PW—policy workers; ^2^ HCW—healthcare workers.

## Data Availability

The data presented in this study are available on request from the corresponding author. The data are not publicly available due to an agreement with the participating organizations. During the consent process, the organizations participating in the MINUTES study were explicitly guaranteed that the data would be pseudonymized by the study’s research center and that the pseudonymized data would only be seen by members of the study team.
